# Comparative Transcriptome Analysis of the Less-Dormant Taiwanese Pear and the Dormant Japanese Pear during Winter Season

**DOI:** 10.1371/journal.pone.0139595

**Published:** 2015-10-09

**Authors:** Yoshihiro Takemura, Katsuou Kuroki, Yoji Shida, Shungo Araki, Yukari Takeuchi, Keisuke Tanaka, Taichiro Ishige, Shunsuke Yajima, Fumio Tamura

**Affiliations:** 1 Faculty of Agriculture, Tottori University, Koyama, Tottori, 680–8553, Japan; 2 NODA Genome Research Center, Tokyo University of Agriculture, Setagaya-ku, Tokyo, 156–8502, Japan; 3 Department of Bioscience, Tokyo University of Agriculture, Setagaya-ku, Tokyo, 156–8502, Japan; National Key Laboratory of Crop Genetic Improvement, CHINA

## Abstract

The flower bud transcriptome in the less dormant Taiwanese pear ‘Hengshanli’ and high-chilling requiring Japanese pear strain TH3 subjected to the same chilling exposure time were analyzed during winter using next-generation sequencing. In buds sampled on January 10th and on February 7th in 2014, 6,978 and 7,096 genes, respectively, were significantly differentially expressed in the TH3 and ‘Hengshanli’ libraries. A comparative GO analysis revealed that oxidation-reduction process (biological process) and ATP binding (molecular function), were overrepresented during the ecodormancy period (EP) when compared to the endodormancy deepest period (DP), indicating that ATP synthesis was activated during the transition between these dormancy stages. Among the 11 differently expressed genes (DEGs) annotated as probable dehydrins or LEA protein-related genes, 9 DEGs showed higher transcript levels in the DP than in the EP. In order to focus on transcription factors induced by low temperature or drought, 7 differently expressed genes (DEGs) annotated as probable *ICE1* or *DREB* proteins were analyzed by real-time PCR. Expression levels of 3 genes were higher in TH3 than in ‘Hengshanli’ on all sampling days. Their expression increased during the endodormancy deepest period (DP) and then decreased before endodormancy breaking in TH3 buds. Taken together, these results suggest that these genes annotated as *ICE1*, *DREB* and *ERF* are involved in endodormancy maintenance and in the transition from endodormancy to ecodormancy.

## Introduction

Bud dormancy in temperate zone deciduous fruit trees is an adaptive mechanism that allows trees to survive under unfavorable conditions during winter [1]. The three dormancy stages are paradormancy, endodormancy, and ecodormancy [2]. In autumn, buds enter endodormancy after defoliation and growth cessation. At this stage, bud growth is not possible even under favorable environmental conditions. Endodormancy breaks after a period of sufficiently low temperatures known as the chilling requirement (CR), the required temperature and duration of the CR depends on the species and cultivar [3]. However, if the CR is not met, such as in periods of climate change or global warming, endodormancy does not break and new organ growth does not occur in spring [4].

Pears (*Pyrus* spp.) are among the most important perennial deciduous fruit trees in the world and Japanese pear (*Pyrus pyrifolia* Nakai) is one of the most important fruits in Japan. Recently, in regions such as New Zealand [5] and Brazil [6] low chilling in winter has resulted in reduced bud breaking in Japanese pears during spring. Thus, elucidating the mechanisms underlying the dormancy stages and endodormancy break in pear trees and other deciduous fruit trees is a prerequisite for developing countermeasures against global warming.

To address this issue, several studies have been conducted to examine endodormancy in various tree species. Water availability is one of the basic factors that determines bud development because it is thought that dormancy is closely related to changes in the water movement, as this is an essential event in the overwintering process of woody plants [7]. The total soluble sugar and water contents accumulation period in peach buds during endodormancy was different in two cultivars with different CRs, even though the species was the same [8]. Moreover, Yooyongwech et al. [8] showed that the expression levels of *Pp-γTIP1* and *Pp-PIP1* genes encoding aquaporin proteins that regulate water transport in the tonoplast and plasma membrane increased in peach buds of high-chilling requiring cultivars compared with those of low-chilling requiring cultivars before the breaking of endodormancy. In addition, bud dormancy has also been related to phytohormone fluctuations. For example, endogenous abscisic acid (ABA) levels induced by low temperature or drought stress increased when endodormancy is established and decreased during endodormancy release in apple [9] and pear [10] buds. Accordingly, after dormancy induction by short days in autumn, ABA signal transduction components (*PP2C* or *AREB3*) were induced in poplar buds [11]. A conceptual model developed for seasonal dormancy transitions in crown buds of leafy spurge based on microarray studies also highlighted the role of *DREB1A/CBF2*, *COP1*, *HY5*, *DELLAs*, *DAM*, *FT* in the maintenance of well-defined dormancy phases [12]. In addition, the cold-induced expression of some *dehydration-responsive element binding protein (DREB)/C-repeat binding factor (CBF)*-family members [13], and *DREB1A* has been identified as a central regulator of molecular networks involved in endodormancy induction [12] and break [14]. A recent study of *P*. *pyrifolia* focused on determining the molecular levels of *MIKC-type dormancy-associated MADS-box* (*DAM*) genes, which may be candidate endodormancy-breaking genes [15]. Expression of *dam* genes decreased during the breaking of endodormancy in the Japanese pear ‘Kosui’ and was very low in the Taiwanese pear (TP-85-119), which is a less dormant pear species [15]. A comparison between ‘Kosui’ and the less dormant Taiwanese pear ‘Hengshanli’ (TP-85-119) identified two novel transcription factors (*NAC* and *PRR*) whose expression levels varied concomitantly with dormancy phase changes [16].

Recently, microarray analysis and RNA sequencing using next-generation sequencing technology (RNA-seq) have widely been used for the transcriptomic analysis of dormancy in plants such as Japanese pear [16], [17], [18], grapevine [19], and Japanese apricot [20]. By comparing buds of the same cultivar exposed to different chilling periods, these studies identified several genes involved in dormancy phase transitions such as stress response-, cell cycle- and phytohormone-related genes. In this study, we analyzed the transcriptome of flower buds exposed to the same chilling period in the less dormant Taiwanese pear ‘Hengshanli’ and high-chilling requiring Japanese pear strain TH3 during winter using next-generation sequencing. By comparing the transcriptome of buds collected on the same date, it is possible to limit the effect of genes showing large diel variation caused by water stress due to limited rain, therefore, we expect to effectively isolate genes expressed specifically during the endodormancy stage. In addition, by comparing buds of two pear strains at different endodormancy states despite their exposure to the same chilling period, we expect to effectively isolate transcription factors induced by low temperature, and are involved in endodormancy maintenance.

## Materials and Methods

### Plant materials and percentage of floral bud break in the 2013–2014 and 2014–2015 seasons

Samples were collected from high-chilling requiring Japanese pear strain TH3, which is bred by selfing ‘Osa-Gold Nijisseiki’, and from the less dormant Taiwanese pear ‘Hengshanli’ grafted onto *P*. *betulaefolia* seedlings in the orchard of Tottori University (35.5° N, 134.2° E), Tottori city, Japan. Lateral floral buds and branches were collected on January 10th and February 15th in the 2013–2014 season, and periodically from December 24th to February 3rd in the 2014–2015 season. The buds from the TH3 strain were considered as T1 if collected on January 10th, 2014, and were considered as T2 from February 7th onwards. The buds from ‘Hengshanli’ trees were labeled as H1 and H2 when sampled on January 10th and February 7th, 2014, respectively. Floral buds were frozen immediately after collection in liquid nitrogen and stored at -80°C until RNA extraction.

In order to determine the percentage of floral bud break, branches of approximately 30 cm long that included five lateral floral buds were used. The basal part of the cuttings was submerged in 0.03% (v/v) aluminum sulfate and 0.3% (v/v) 8-hydroxyquinoline. The cuttings were then maintained in a growth chamber at 23 ± 1°C and 24-h photoperiod. Bud break is defined as a developmental stage of more than four phases characterized by swelling of the buds and the emergence of a green tip between scales. Bud break percentage was determined 14 d after forcing on five single shoots with five buds. Chilling unit (CU) values were calculated using the Saitama method [21] as described by Tamura et al. [22].

### RNA extraction, library preparation, and RNA-seq

Total RNA was extracted from 3 biological replicates of buds collected on each sampling date according to the methods described in Gasic et al. [23] with some modifications. An independent pool for transcriptome analyses was produced from buds in T1, T2, H1 and H2. Genomic DNA was eliminated from the total RNA preparation using DNase I (New England Biolabs Inc., Ipswich, MA, USA). The quality of total RNA was evaluated with an Agilent 2100 Bioanalyzer using an Agilent RNA 6000 nano Kit (Agilent Technologies, Santa Clara, CA, USA). Only the samples with a RNA integrity number (RIN) > 8.0 were used for RNA-seq. Library preparation was performed according to the TruSeq RNA Sample Preparation v2 guide (Illumina, San Diego, CA, USA). Oligo-(dT) magnetic beads were used to isolate poly-(A) mRNA from total RNA, and fragmentation buffer was added to cut mRNA into short fragments. Using these short fragments as templates, first-strand cDNAs were synthesized using random hexamer-primers and SuperScript II Reverse Transcriptase (Invitrogen). After second-strand cDNA synthesis, end-repaired and dA-tailed fragments were connected with sequencing adaptors. The adapter-ligated cDNA fragments were amplified by 15 cycles of PCR, and the products were cleaned up using AMPure XP magnetic beads (Beckman Coulter, Pasadena, CA, USA). Library quality and concentration were assessed using an Agilent Bioanalyzer 2100 and an Agilent DNA 1000 kit (Agilent Technologies, Santa Clara, CA, USA). The concentration of the libraries was more precisely determined by quantitative real-time PCR using a KAPA Library Quantification Kit (Kapa Biosystems).

All libraries were first diluted to a concentration of 10 nM and mixed in equal amounts. After denaturalization with 0.2 N NaOH, the final concentration of the library mixture was diluted to 13 pM including 1% PhiX library (Illumina, San Diego, CA, USA). The library mixture was sequenced by 2 × 100 bp paired-end sequencing using an Illumina HiSeq 2500 (Illumina, San Diego, CA, USA). Reads in FASTQ format were generated using an Illumina Casava pipeline (version 1.8.3). The read data were submitted to the DDBJ Read Archive (Accession number DRA003270).

### De novo assembly and clustering analysis

Raw reads containing adaptors were removed using TagDust (version 1.13). Additionally, FASTX-Toolkit (version 0.0.13.2) was used to trim the first 13 bp of each read, to clip uncertain bases appearing as “N”, and to filter reads based on their quality scores. Quality filtering parameters were as follows: (i) Minimum quality score to keep, 20; and (ii) Minimum percent of bases that must have [-q] quality, 80. Among the remaining reads, unpaired-reads were then removed using a custom Perl script. After combining the analysis data in twice on each sample, *de novo* assemblies were performed using Trinity (version r20140413) using the default settings. Highly similar contigs were clustered using CD-HIT (version 4.3) and TGICL (version 2.1).

### Statistical analysis of expression levels and homology search

Using CLC Genomics Workbench 7.0.4 (Qiagen), read data without adaptors and with the first 13 bp trimmed were mapped to the references that consist of the clustered contigs. Mapping parameters were as follows: (i) Mismatch cost, 2; (ii) Insertion cost, 3; (iii) Deletion cost, 3; (iv) Length fraction, 0.5; and (v) Similarity fraction, 0.95. To compare expression levels between two samples (T1 vs H1, T2 vs H2, and H1 vs H2), mapping-based count data was constructed from "Total counts" and was tested using edgeR. Differentially expressed genes (DEGs) or common genes between samples were selected according to the false discovery rate (FDR, q-value < 0.05). All references clustered by CD-HIT and TGICL programs were aligned by BLASTx (E-value < 1e-7) to the NCBI non-redundant protein database and annotated according to the top hit description. "Total counts" of genes with the same annotation information were combined, and genes that showed the same expression pattern between two samples in both CD-HIT and TGICL analyses were defined as DEGs. To select DEGs related to the endodormancy stage transition, the DEGs obtained by comparing H1 vs H2 were subtracted from each of the DEGs obtained by comparing T1 vs H1 and T2 vs H2. DEGs that showed higher expression levels in T1 and H1 libraries were annotated using Blast2GO [24] plugin in the CLC Genomics Workbench with Gene Ontology (GO) [25].

### Real-time PCR

Total RNA was isolated from the buds and used to synthesize first-strand cDNAs with reverse transcriptase (TaKaRa). cDNA was diluted 1/100 and used as a template for real-time PCR. Real-time PCR was performed using the SYBR Green system on a LightCycler 480 (Roche Diagnostics, Basel, Switzerland). A total of 5 μL of diluted cDNA was added to 15 μL of the reaction mixture containing 3 μL of LightCycler 480 SYBR Green Mastermix and 0.5 mM of specific primer pairs for each gene (S1 Table). Specific primers for each gene were designed using the Primer Express software. Real-time PCR was performed in the following manner: an initial step of 2 min at 95°C was carried out, followed by 45 cycles of 10 s at 95°C, 20 s at 60°C, and 20 s at 72°C, melting for 0 s at 95°C, and slow heating from 60°C to 95°C at 0.2°C/s to confirm the amplification of single products. The specificity of the amplification reaction for a given primer set was verified on the basis of the melting curves. Relative expression was determined using the 2^-ΔΔCt^ algorithm and normalized to the actin gene [26] which did not show differential expression in this study.

## Results

### Dormancy status of ‘Hengshanli’ and TH3 in the 2013–2014 and 2014–2015 seasons

In all sampling date of both seasons, leaves of TH3 and ‘Hengshanli’ had already been defoliated. In the 2013–2014 season, the percentage of floral bud break in TH3 increased from 0% to 32% from January 10th to February 7th (Table 1). In contrast, the percentage of floral bud break in ‘Hengshanli’ remained constant at 100%. Based on the percentage of floral bud break, TH3 buds were in the deepest period (DP) phase of endodormancy on January 10th, 2014, and the buds then transitioned to the breaking period (BP) phase on February 7th, while the ‘Hengshanli’ buds were in the ecodormancy period (EP) on both sampling dates in 2014.

**Table 1:** Floral bud break (%) of TH3 and ‘Hengshanli’ in 2013–2014 season.

Date (CU ^Z^)	Cultivar	Bud break (%) ^Y^	Dormancy stage	Sample No.
10—Jan (1134)	TH3	0.0 c ^X^	Endodormancy deepest period (DP)	T1
	Hengshanli	100.0 a	Ecodormancy period (EP)	H1
7—Feb. (1731)	TH3	32.0 b	Endodormancy breaking period (BP)	T2
	Hengshanli	100.0 a	Ecodormancy period (EP)	H2

^Z^ CU value at sampled date was calculated from 31 Oct.

^Y^ 14 days after forcing.

^X^ Different letters within the same column show a significant difference at *P*<0.05 by t-test.

In the 2014–2015 season, the percentage of floral bud break in TH3 was 0% from December 24th to January 20th, which then increased to 64% on February 3rd (Table 2). The percentage of floral bud break in ‘Hengshanli’ was over 85% on all sampling dates.

**Table 2:** Seasonal changes in percent floral budbreak of *Pyrus* plants in 2014–2015 season.

Registered name (Cultivar or lines)		Budbreak (%) ^Z^			
	Date	24—Dec.	8—Jan.	20—Jan.	3—Feb.
	CU ^Y^	767	1111	1394	1709
TH3		0 b ^X^	0 b	0 b	64 a
Hengshanli		88 a	96 a	100 a	96 a

^Z^ 14 days after forcing.

^Y^ CU value at sampled date was calculated from 31 Oct.

^X^ Mean separation within lows by t-test at *P*<0.05.

### Sequencing, de novo assembly, and clustering analysis

RNA isolated from TH3 and ‘Hengshanli’ floral buds collected on January 10th and February 7th, 2014, was used to synthesize the cDNA for paired-end sequencing using the Illumina HiSeq 2500. The RNA-seq generated a total of 0.5 billion reads from each sample and a total of 870,440 contigs were assembled using clean reads (S2 Table). By clustering analysis using CD-HIT and TGICL programs, 314,420 and 65,551 contigs were assembled, respectively. All contigs generated by TGICL were defined as unigenes in this study. The percentage of reads mapped using the two clustering programs in each library ranged from 65.1–84.0%.

### Genes showed differential expression between dormancy stages

By subtracting the DEGs obtained by comparing H1 vs H2 from each of the DEGs obtained by comparing T1 vs H1 and T2 vs H2 libraries, DEGs related to the endodormancy stage transition were identified in the two comparative analyses, i.e., in the T1 vs H1 and T2 vs H2 comparisons (S3 Table). In flower buds sampled on January 10th, 6,978 genes were significantly differentially expressed in the T1 and H1 libraries. Of these, 2,756 and 4,222 were DEGs with higher expression levels in the T1 and H1 libraries, respectively. When comparing T2 and H2 libraries, 7,096 DEGs were identified of which 2,531 and 4,565 had higher expression levels in T2 and H2, respectively.

**Fig 1:**
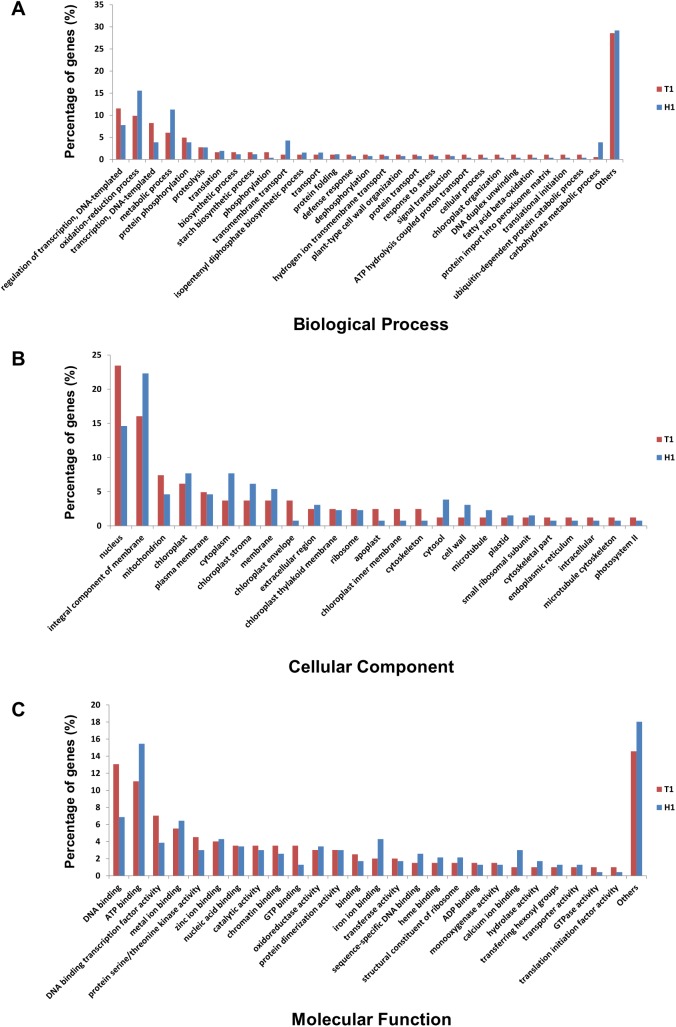
Functional categories of DEG genes in the Gene Ontology. GO categories were analyzed in pairwise comparisons in between T1 and H1 libraries. DEG genes were classified by biological process (A), cellular component (B) and molecular function (C).

To compare function of DEGs in the different dormancy statuses, DEGs that showed higher expression levels in the T1 and H1 libraries were annotated using Blast2GO. A comparative GO analysis considering biological processes showed that genes involved in oxidation-reduction metabolic, and carbohydrate metabolic processes, and transmembrane transport were present in a higher percentage in the H1 than in the T1 library (Fig 1(A)). More than 20% of the genes were shown to be nuclear and integral membrane components in the T1 and H1 libraries, respectively, when DEGs were classified according to their cellular component (Fig 1(B)). Classification by molecular function showed that a higher percentage of DEGs was involve in DNA binding in T1 than in H1, while a higher percentage of DEGs was involve in ATP binding in H1 than in T1 (Fig 1(C)).

### Transcriptome analysis of DEGs

**Fig 2:**
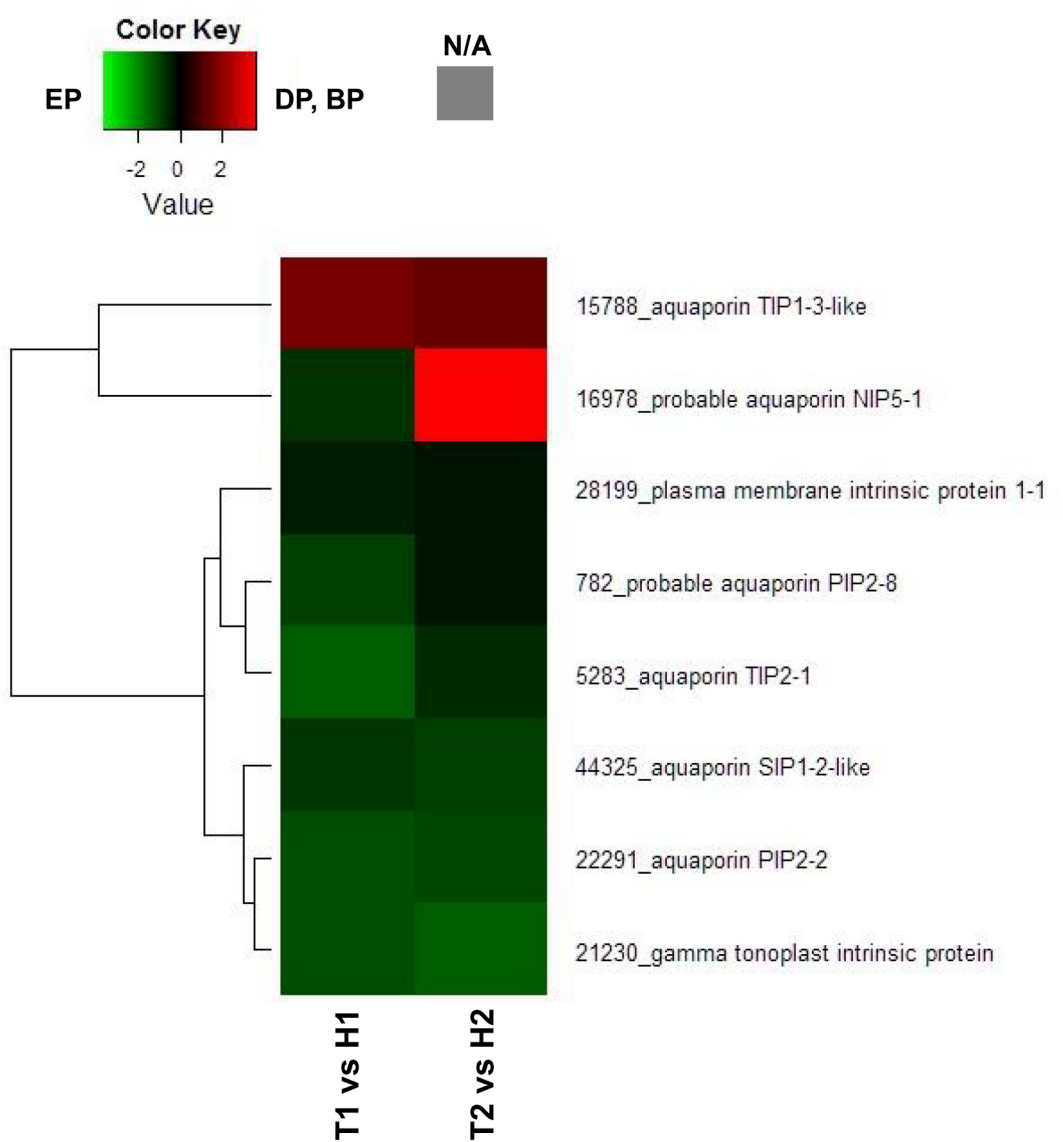
Heat map diagram of relative gene expression levels for DEGs annotated as probable aquaporin-related genes. Red indicates a relative higher levels in expression in DP or BP than EP, and green represents a relative higher levels in expression in EP than DP or BP.

**Fig 3:**
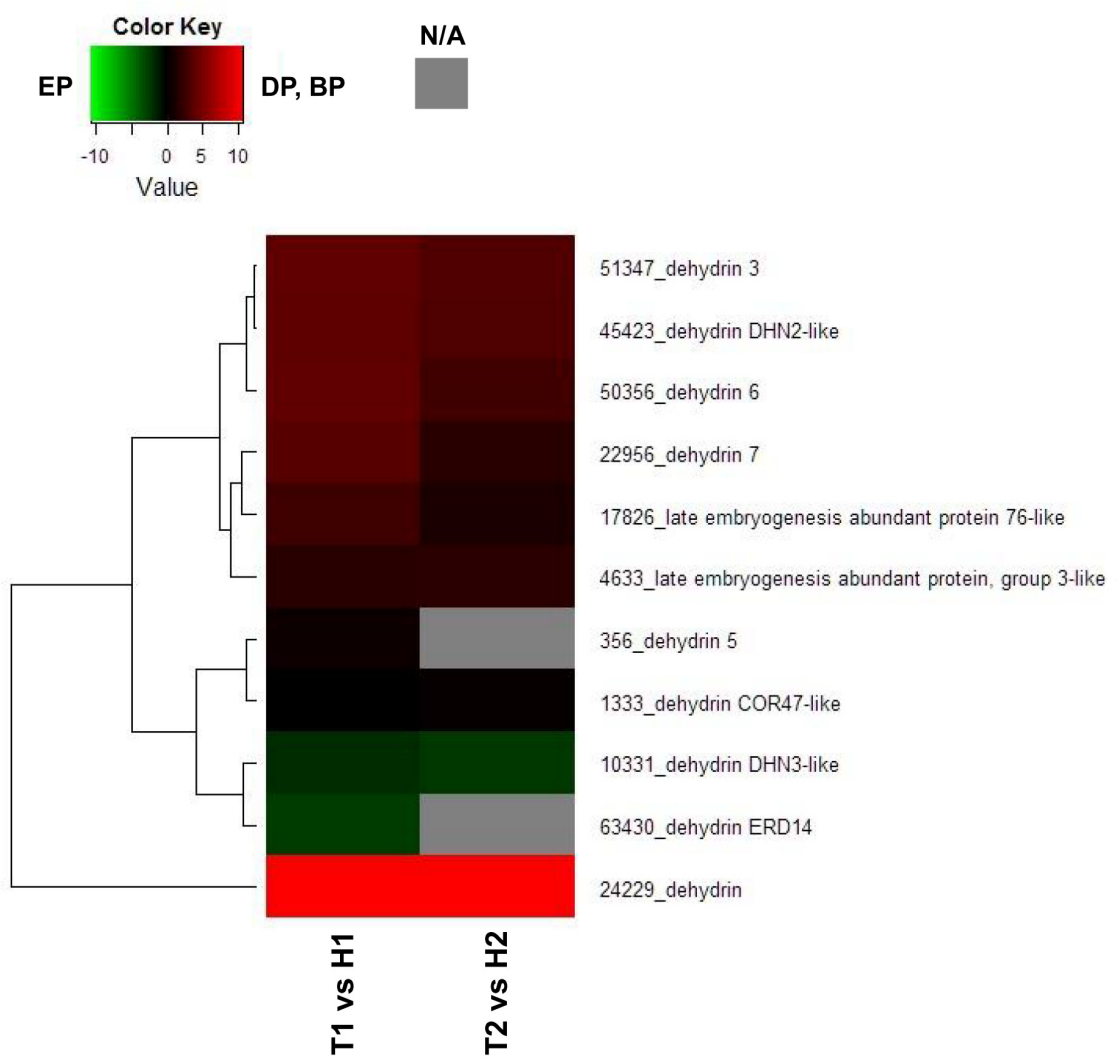
Heat map diagram of relative gene expression levels for DEGs annotated as probable dehydrin or LEA protein-related genes. Red indicates a relative higher levels in expression in DP or BP than EP, and green represents a relative higher levels in expression in EP than DP or BP.

Endodormancy is closely related to changes in the water movement, many reports have shown that aquaporin, dehydrin or LEA protein involved in regulation of endodormancy [8] [27] [28]. To serve as representative examples, we constructed heat map diagrams of relative gene expression levels for DEGs annotated as probable aquaporin-, dehydrin- or LEA-related genes. Among the 8 DEGs in the T1 vs H1 comparison annotated as probable aquaporin-related genes, 7 DEGs showed higher transcript levels in the EP (H1) than in the DP (T1) and 6 DEGs showed higher levels in the EP (H2) than in the BP (T2) (Fig 2). In contrast, among the 11 DEGs in the T1 vs H1 comparison annotated as probable dehydrin- or LEA protein-related genes, 9 DEGs showed higher transcript levels in the DP (T1) than in the EP (H1) (Fig 3).

**Fig 4:**
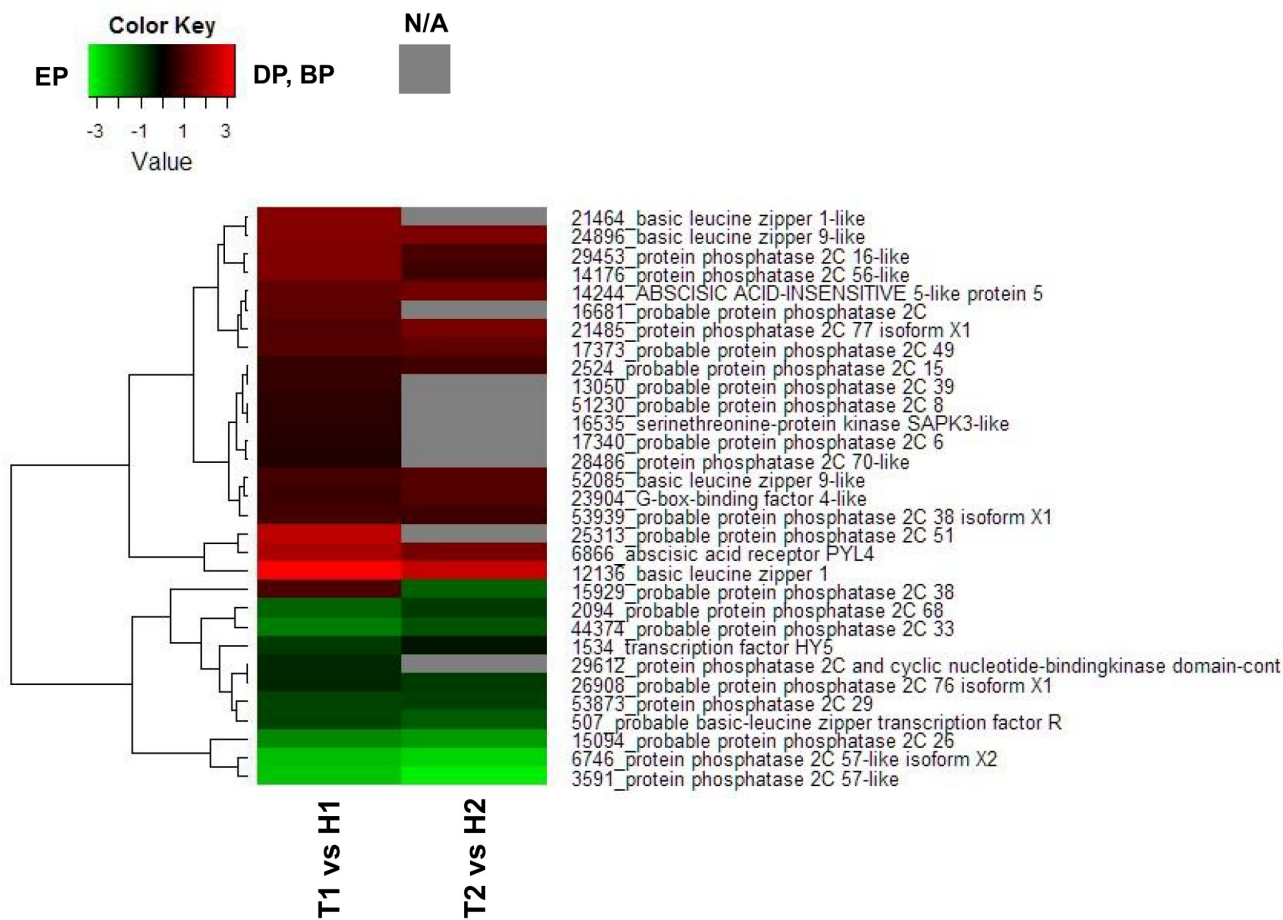
Heat map diagram of relative gene expression levels for DEGs annotated in signal transduction pathways of ABA. Red indicates a relative higher levels in expression in DP or BP than EP, and green represents a relative higher levels in expression in EP than DP or BP.

To investigate the relationship between phytohormone signaling and the transition between the different dormancy stages, DEGs related to ABA and gibberellin (GA) signaling pathways were analyzed. Among the 31 DEGs identified as related to the ABA signal transduction pathway, 21 DEGs were annotated as probable protein phosphatase 2C (Fig 4). All of the 4 DEGs annotated as probable basic leucine zipper genes showed higher transcript levels in the DP (T1) than in the EP (H1). In the ABA signaling pathway heat map diagram from the T2 vs H2 comparison, transcript levels of 10 DEGs were higher in the EP (H2) than in the BP (T2), and 12 DEGs showed higher levels in the BP (T2) than in the EP (H2).

**Fig 5:**
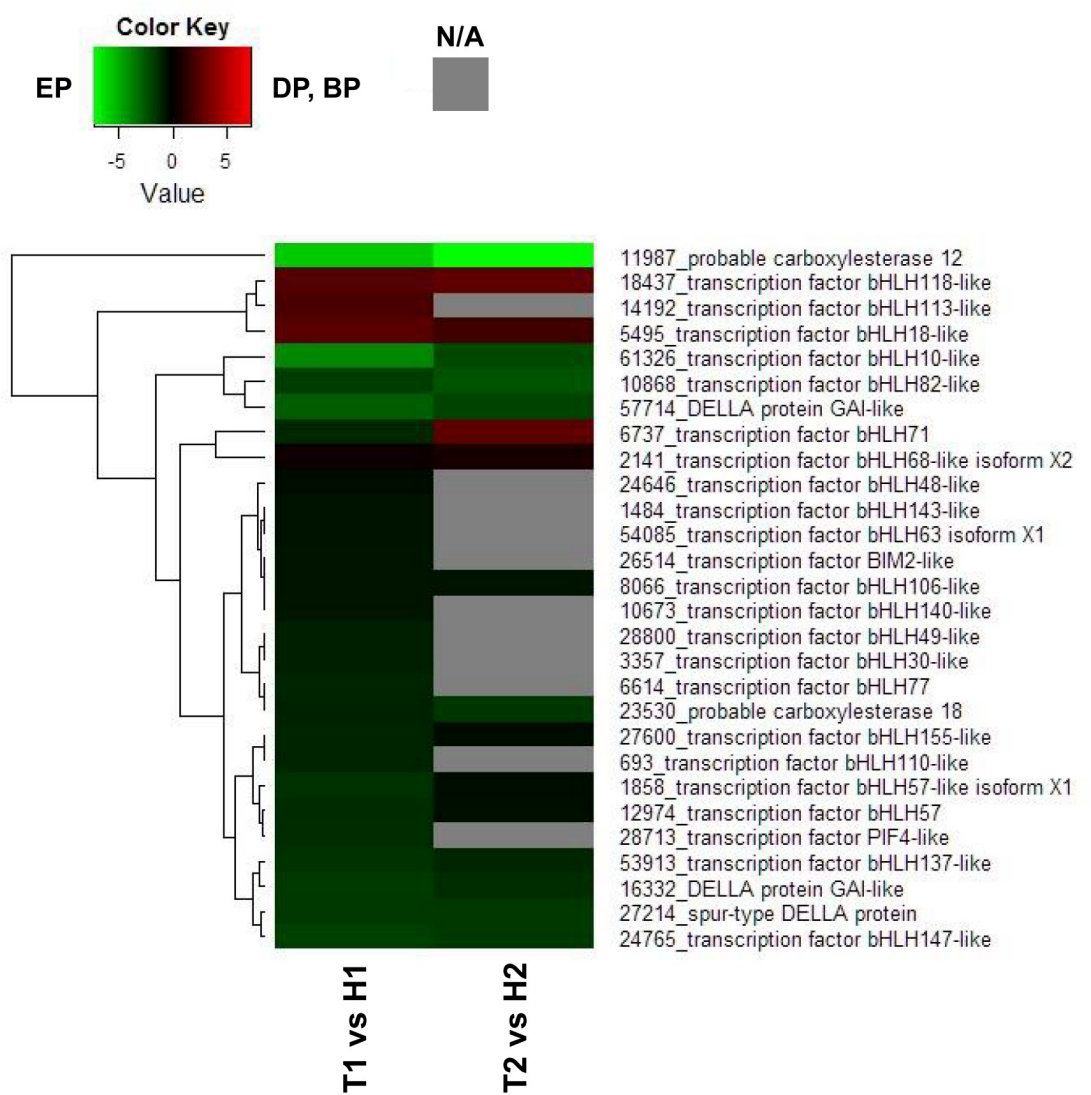
Heat map diagram of relative gene expression levels for DEGs annotated in signal transduction pathways of GA. Red indicates a relative higher levels in expression in DP or BP than EP, and green represents a relative higher levels in expression in EP than DP or BP.

In the GA signal transduction pathway, among the 22 DEGs annotated as probable bHLH transcription factors, the expression of 18 DEGs was higher in the EP (H1) than in the DP (T1) (Fig 5). In the GA signaling pathway heat map diagram from the T2 vs H2 comparison, transcript levels of 10 DEGs were higher in the EP (H2) than in the BP (T2). In contrast, 12 DEGs showed higher expression levels in the BP (T2) than in the EP (H2).

**Fig 6:**
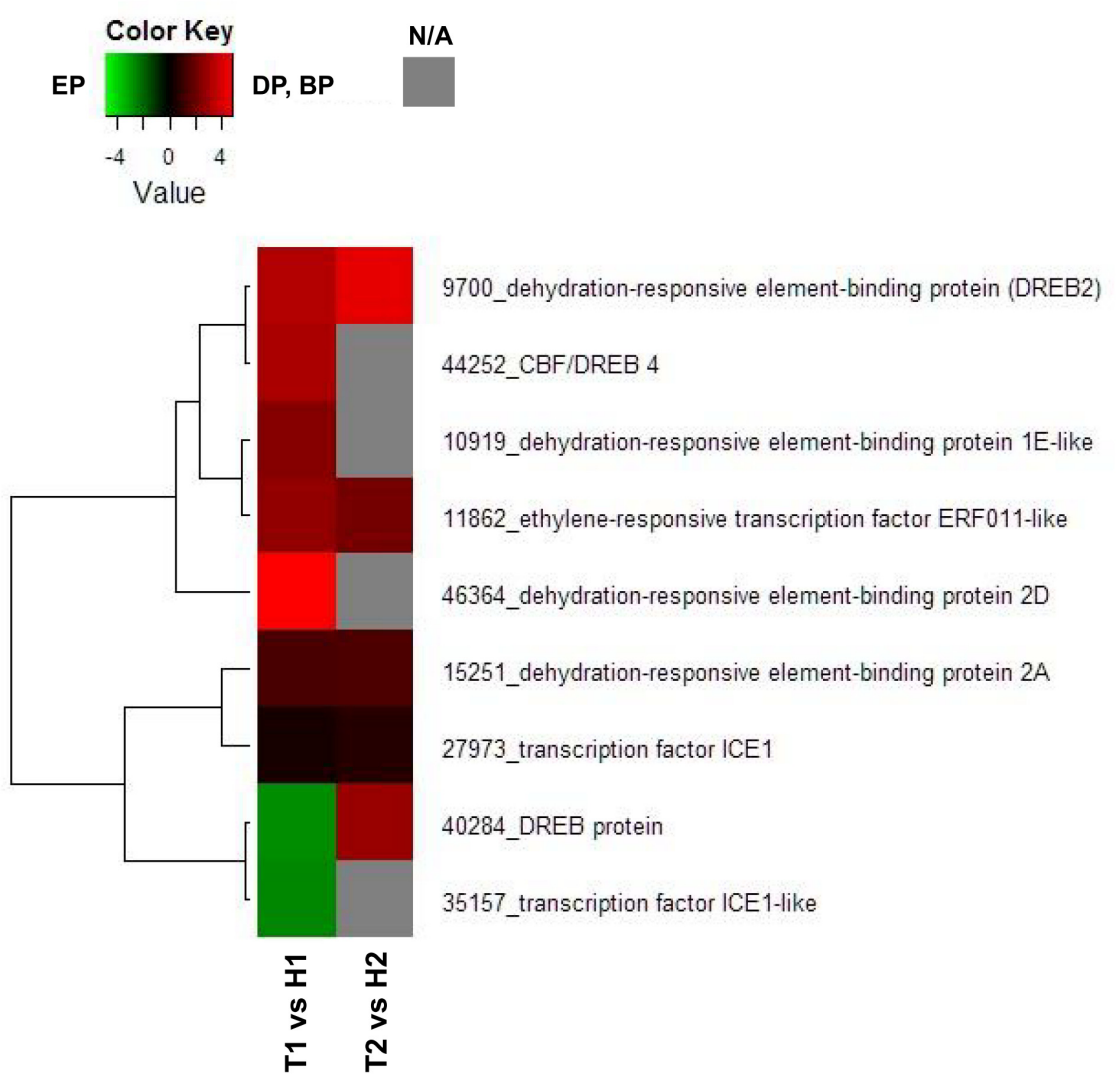
Heat map diagram of relative gene expression levels for DEGs annotated as probable ICE or DREB-related genes. Red indicates a relative higher levels in expression in DP or BP than EP, and green represents a relative higher levels in expression in EP than DP or BP.

To focus on the DEGs annotated as probable *ICE1* or *DREB*, we constructed a heat map diagram of relative gene expression levels (Fig 6). Among all of the 9 DEGs identified as probable *ICE1* or *DREB*, transcript levels of 7 DEGs were higher in the DP (T1) than in the EP (H1), while no DEG showed higher expression levels in the EP (H2) than in the BP (T2).

### Expression analysis of DEGs related to *bZIP* and *WRKY* in ‘Hengshanli’ and TH3 by Real-time PCR

**Fig 7:**
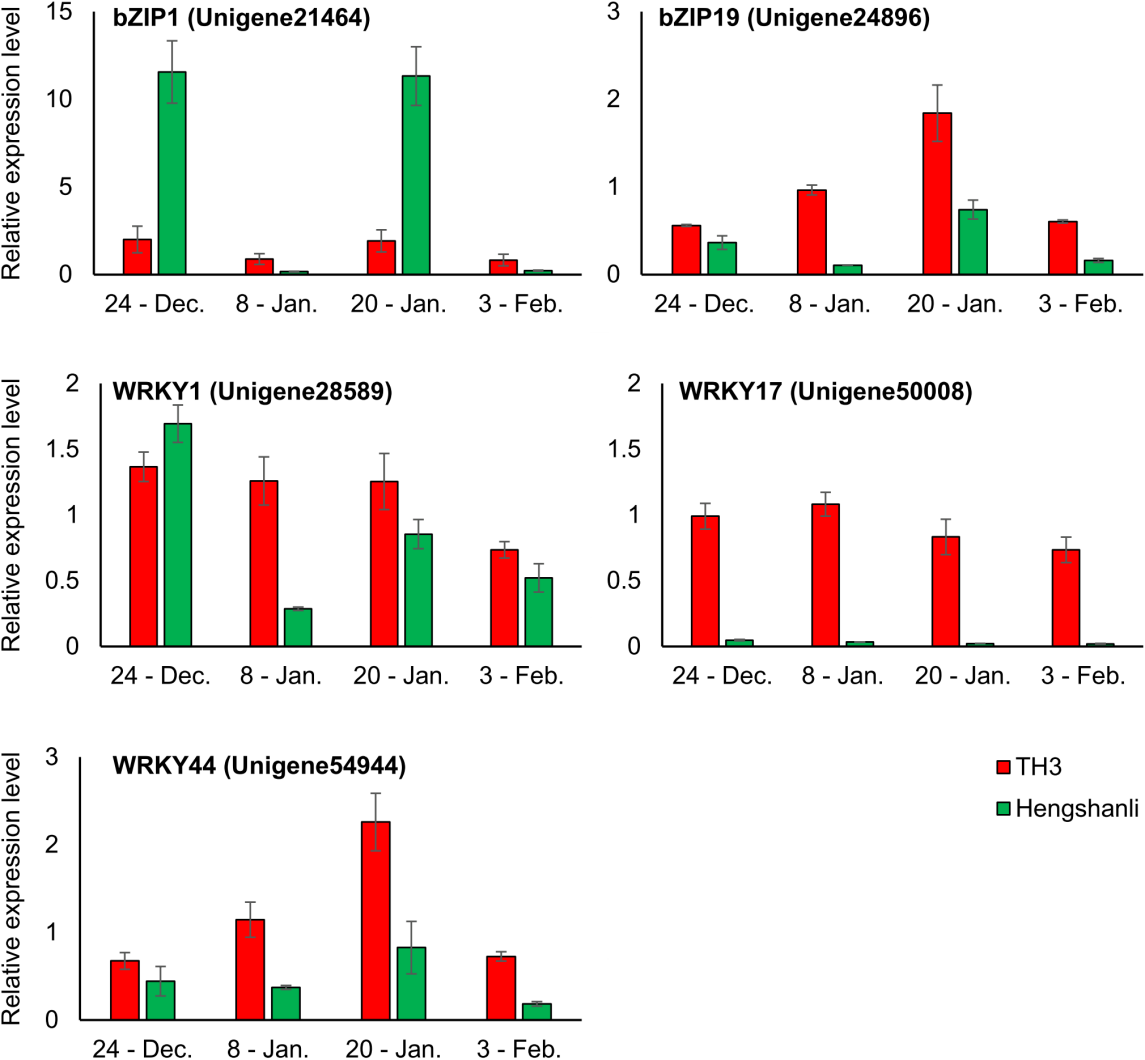
Relative expression levels of genes annotated as *bZIP* and *WRKY* in TH3 and ‘Hengshanli’ during winter season (2014–2015).

*bZIP* and *WRKY* are two important plant transcription factor families regulating diverse developmental and stress-related processes [29]. DEGs of 2 types annotated as probable *bZIP* in signal transduction pathways of ABA and DEGs of 3 types annotated as probable *WRKY* were higher in the DP (T1) than in the EP (H1) and these DEGs were analyzed using real-time PCR. In the expression analysis carried out in TH3 and ‘Hengshanli’ floral buds collected from December 24th to February 3rd in the 2014–2015 season, expression levels of 5 DEGs on January 8th were higher in TH3 than in ‘Hengshanli’ (Fig 7). Expression levels of *bZIP1* (Unigene21464) on December 24th and January 20th and *WRKY1* (Unigene28589) on December 24th were higher in ‘Hengshanli’ than in TH3. On the other hands, expression levels of *bZIP19* (Unigene24896) and *WRKY* of 2 types (Unigene50008 and Unigene54944) from December 24th to February 3rd were higher in TH3 than in ‘Hengshanli’. Additionally, expression levels in TH3 of the three unigenes decreased from January 8th to January 20th and from January 20th to February 3rd.

### Expression analysis of DEGs related to *ICE1* and *DREB* in ‘Hengshanli’ and TH3 by Real-time PCR

**Fig 8:**
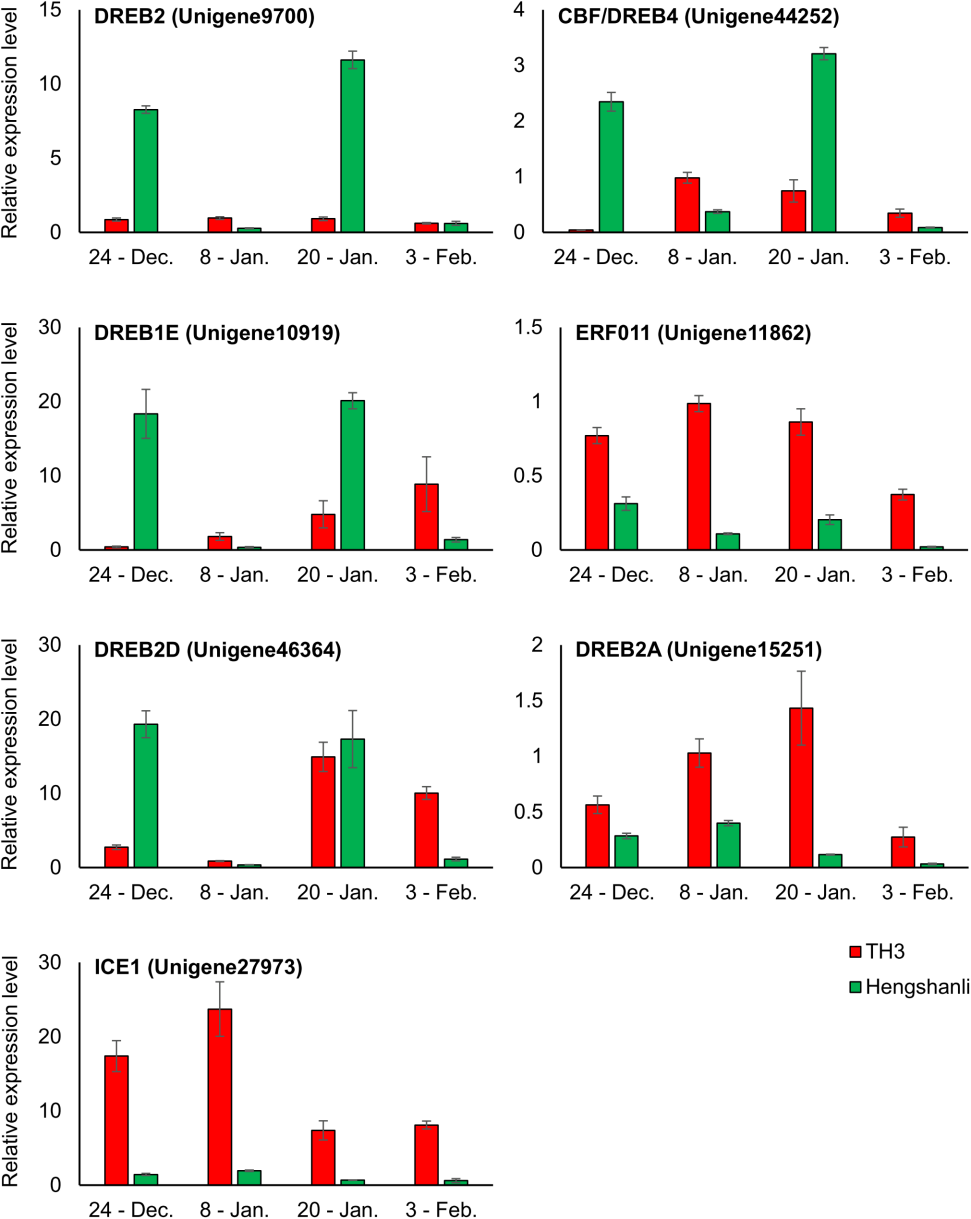
Relative expression levels of genes annotated as *ICE1* and *DREB* in TH3 and ‘Hengshanli’ during winter season (2014–2015).

*ICE1* or *DREB* genes were also proposed to be involved in the endodormancy maintenance [13], [14], [30]. Among the 9 DEGs annotated as probable *ICE1* or *DREB*, expression levels of 7 DEGs were higher in the DP (T1) than in the EP (H1) and these DEGs were analyzed using real-time PCR. In the expression analysis carried out in TH3 and ‘Hengshanli’ floral buds collected from December 24th to February 3rd in the 2014–2015 season, expression levels of all 7 genes on January 8th were higher in TH3 than in ‘Hengshanli’ (Fig 8). The 7 genes were classified into 2 subgroups based on their expression patterns: type 1 genes included those genes which expression levels on December 24th and January 20th were higher in ‘Hengshanli’ than in TH3 (Unigene9700, Unigene44252, Unigene10919, and Unigene46364), and type 2 genes included those genes which expression levels from December 24th to February 3rd were higher in TH3 than in ‘Hengshanli’ (Unigene11862, Unigene15251, and Unigene27973). Expression levels in TH3 of the three type 2 unigenes increased from December 24th to January 8th, while it decreased from January 8th to January 20th and from January 20th to February 3rd.

## Discussion

Endodormancy in temperate zone deciduous fruit trees is a strongly regulated strategy to survive environmental extremes. In order to monitor gene expression changes during winter dormancy, Bai et al. [17] and Liu et al. [18] compared the transcriptomes of pear buds on different sampling dates during the transition from endodormancy to ecodormancy in the same cultivar using RNA-seq. In the present study, in order to uncover the biochemical processes involved in endodormancy maintenance and to identify the transcription factors related to this maintenance, we analyzed floral bud transcriptome of two pear strains that show different dormancy state at the same sampling date.

From the results obtained in the 2013–2014 season regarding the percentage of floral bud break, we can conclude that the dormancy state on January 10th and February 7th varies widely between TH3 and ‘Hengshanli’ trees. Leaves of ‘Hengshanli’ had already been defoliated at both sampling dates, in addition, the dormancy of ‘Hengshanli’ had already progressed to an ecodormancy stage by those sampling dates. Therefore, we performed RNA-seq using these floral buds and then subtracted DEGs obtained by comparing H1 vs H2 from each of the DEGs obtained by comparing T1 vs H1 and T2 vs H2 to exclude those DEGs that are not involved in the transition between dormancy stages. Later, we performed a comparative GO analysis of the DEGs identified when comparing the T1 (DP) and H1 (EP) libraries in order to determine the different functions of DEGs in the different dormancy stages. The GO analysis results suggest that DEGs contain genes involved in stage-specific expression at each dormancy stage. A comparative GO analysis considering the biological process and molecular function detected that the percentage of genes related to the oxidation-reduction process and ATP binding were higher in the H1 than in the T1 library. In grapes, it has been shown that hydrogen cyanamide (HC) and heat shock treatment can induce endodormancy breaking, which is followed by the activation of the tricarboxylic acid cycle (TCA cycle), ATP synthesis, and oxidative phosphorylation involved in the oxidation-reduction process [31]. Recent microarray results in tree peony indicate that the ATP-binding cassette (ABC transporter) family protein was down-regulated during the endodormancy stage, while it was upregulated during the ecodormancy stage [32]. From these results, it was gathered that buds on ecodormancy stage, still unable to grow, have already commenced the ATP synthesis necessary for growth.

Results of comparative GO analysis considering the cellular component also indicated that a higher percentage of DEGs were integral membrane components in the H1 than in the T1 library. Aquaporins, known as water channel proteins, are intrinsic membrane proteins that constitute a major route for water transport across the cell membrane [33]. Therefore, we focused on aquaporins and constructed a heat map diagram of relative gene expression levels for DEGs annotated as probable aquaporins. Among the 8 genes annotated as aquaporins, 7 showed higher transcript levels in the EP (H1 and H2). In peach flower buds, the expression of *TIP1* and *PIP2* aquaporins increased earlier in the low-chill cultivar ‘Coral’ than in the high-chill cultivar ‘Kansuke Hakuto,’ reflecting the difference in timing for the end of endodormancy in the two cultivars [34]. Endodormancy is closely related to changes in water movement, hence, aquaporins are required for bud activity at the end of endodormancy, resulting in a gradual increase in water content.

Moreover, these results show that transcripts of genes encoding late embryogenesis abundant (*LEA*) protein or dehydrin (related to drought stress) are significantly higher in the DP. Dehydrins, such as *LEA* proteins, were identified as being associated with dormancy transition [31]. Dehydrins, known as a multi-family of proteins produced in response to cold and drought stress, are associated with cold hardiness and with the water content/state in the tissues of trees on endodormancy stage [32]. The expression of some dehydrin genes in Norway spruce (*Betula pubescens* Ehrh.) gradually decreases when approaching bud burst [35]. Additionally, dehydrins are known to be synthesized by cells in response to ABA [36].

ABA has been termed as ‘dormin’ or ‘dormancy inductor’ [37], and is proposed to promote and maintain bud dormancy in woody plants [38]. During the endodormancy period, it has been considered that ABA accumulation in Japanese pear buds controls endodormancy development [39]. Later, the ABA content decreases during the transition from endodormancy to ecodormancy in leafy spurge [38] and pear buds [10], [17]. In agreement with these findings, our study showed an abundance of DEGs involved in the ABA signaling pathway which had higher expression levels in the T1 library of the DP than in the H1 library of the EP. Genes annotated as protein phosphatase 2C (*PP2C*) were differentially expressed between the two libraries, while the transcript abundance of a gene annotated as a serine/threonine-protein kinase (*SnRK2*) was higher in the DP. ABA binding to the *PP2C* complex allows SnRK2 activation, which then activates downstream transcription factors that induce ABA-responsive gene expression [40]. In agreement with the role suggested for ABA in seed dormancy, the triple mutant *snrk2*.*2 snrk2*.*6 snrk2*.*3* also exhibited loss of dormancy [41]. In addition, the transcript levels of *SnRK2* and ABA-responsive element/ABA binding factor (*AREB/ABF*) genes identified in grapevine were significantly down-regulated in response to HC, which is known as an agricultural chemical for artificial dormancy release [42]. In this study, the transcript abundance of genes annotated as *ABF*s (Unigene14244 and Unigene23904), downstream transcription factors of *SnRK2*, was also higher in the DP (T1) and BP (T2). In addition, we focused on the 5 unigenes annotated as *bZIP* and *WRKY* that showed higher transcript levels in the DP (T1). *bZIP* and *WRKY* are two important plant transcription factor families regulating diverse developmental and stress-related processes, involved in ABA and stress signaling as the transcription factors functioning downstream of *SnRK2* [41]. The real-time PCR results show that expression levels of *bZIP19* (Unigene24896) and *WRKY* of 2 types (Unigene50008 and Unigene54944) from December 24th to February 3rd were higher in TH3 than in ‘Hengshanli’, and decreased from January 8th to January 20th and from January 20th to February 3rd. Moreover, it is interesting to note that expression levels of *WRKY17* (Unigene50008) in ‘Hengshanli’ of EP stage in all sampling days was significantly lower than in TH3. Taken together, these results suggest that ABA signaling pathway activation and higher expression level of transcription factors related to these signaling are necessary to maintain endodormancy.

In contrast to these DEGs involved in the ABA signaling pathway, transcripts of three genes encoding DELLA proteins, found in the GA responsive pathway, were significantly higher in the EP. In the presence of bioactive GAs, DELLAs are generally targeted for degradation leading to the release of growth promoting transcription factors [43]. Taken together, these results proposed that transcription factors induced by low temperature or drought, and ABA or GA are closely involved in the transition from endodormancy to ecodormancy.

In addition to the transcription factor related to ABA or GA, several specific transcription factors and regulatory genes have been demonstrated to play an important role in one or more of the processes in the transition of endodormancy. Among genetic components contributed to the intricate regulation of dormancy, *DAM* genes have been reported to be directly associated with the regulation of dormancy onset and release in peach [44], pear [16], apricot [45] and apple [46], as well the herbaceous plant, leafy spurge [47]. In a previous study, *PpMADS13-1* and *-2* isolated genes from Japanese pear appear to be up-regulated towards endodormancy establishment and down-regulated concomitant with endodormancy release [16], [48], similar to peach *DAM5* and *DAM6* genes [49]. In poplar, the expression of an *EARLY BUD-BREAK1* (*EBB1*) gene plays a major role in regulating the timing of bud break [50]. In addition, the CBF is thought to be an upstream trans-factor regulating *DAM* genes in peach [51], leafy spurge [47] and Japanese pear [48]. A recent report showed that constitutive over-expression of a peach *CBF1* in apple results induction of dormancy by short photoperiod, delayed budbreak and altered expression of several key genes including *DAM* and *EBB*, provide further evidence for *DREB*s playing a role in induce and breaking processes leading to endodormancy [52]. *DREB* or *CBF* genes appear to encode key transcription factors from the major transcription cascade that responds to low temperature and drought [53], [54]. *ICE1*, which is induced by low temperature, enhances *DREB/CBF* gene expression by binding to their promoter regions [55], [56]. Therefore, among the 9 genes annotated as *ICE1* and *DREB* genes in DEGs, we focused on the 7 unigenes that showed higher transcript levels in the DP (T1). The real-time PCR results show that expression levels of all 7 genes on January 8th in the 2014–2015 season were higher in TH3 than in ‘Hengshanli’. These results indicate that expression levels of these genes show little yearly variation, because the expression pattern obtained by real-time PCR matched that obtained by RNA-seq using buds collected on January 10th (CU. 1134) in the 2013–2014 season, which has a similar CU value to January 8th (CU. 1111) in the 2014–2015. Among the 7 unigenes, the expression levels of 3 genes (Unigene11862, Unigene15251, and Unigene27973) analyzed by real-time PCR were always higher in TH3 than in ‘Hengshanli’ in all sampling days. Moreover, it is interesting to note that expression levels of these 3 unigenes in TH3 increased from December 24th to January 8th and then decreased before the BP. Unigene11862, Unigene15251, and Unigene27973 were annotated as *ERF*, *DREB2A* and *ICE1*, respectively. *DREB*s belong to the *ERF* family of transcription factors and are a subfamily of the larger *APETALA2 (AP2)/ERF* superfamily containing involved in abiotic and biotic stress signaling, which has been extensively reviewed [42], [57], [58]. *ICE1* is a *MYC-like bHLH* transcription factor inducing expression of a gene that codes for *CBF/DREB*, a member of the *ERF* family, and also regulates the expression of downstream coldresponsive gene contributing to cold acclimation [59]. The *DREB* and *ERF* sub-families are responsive to ethylene and previous reports have suggested that a transient spike in ethylene may be a pre-requisite to induction of endodormancy by inducing *9-cis-epoxycarotenoid dioxygenase* (*NCED1*), a key regulator in the biosynthesis of ABA, these findings are consistent with several models indicating that metabolic pathways involved in endodormancy induction in leafy spurge [13], [14], [30]. Results in TH3 in expression analysis also suggest that expression of these unigenes annotated as *ICE1*, *ERF* and *DREB* is up-regulated not only by chilling exposure, but also down-regulated by adequate chilling accumulation for bud breaking. Recent study have shown that the presence of up to four *CRT/DRE* motifs in the ~1000 bp 5’ upstream region of the *PpMADS13-1* genes in Japanese pear [48]. The presence of *CRT/DRE* motifs in promoters enhances or modulates target gene expression by DNA-binding proteins belonging to the *AP2/ERF* family, such as *CBF* or *DREB* [60]. The presence of these transcription binding sites was also reported in the putative promoter regions of leafy spurge and peach *DAM* genes [47] [49]. Additionally, *DAM* genes were also proposed to be involved in the endodormacy phase transition in many species, including in peach [44], pear [16], apricot [45] and apple [46]. Taken together, the results in this study suggest that these unigenes involved in endodormancy maintenance and in the transition from endodormancy to ecodormancy.

## Supporting Information

S1 Table Primers used for real-time PCR. (DOCX)

S2 Table Summary of the sequencing, assembly and mapping. ^Z^ 12 libraries as biological replicates constructed from the floral buds of each sample number. Number of replicates shown in brackets.
(DOCX)

S3 Table List of all the differentially expressed genes between T1 and H1, T2 and H2. (XLSX)

## Abbreviations

AREB: ABA-responsive element binding factor
bZIP: basic region/leucine zipper
CBF: C-repeat binding factor
COP1: Constitutive photomorpho- genesis 1
DAM: dormancy-associated MADS-box
DREB: dehydration-responsive element binding protein
ERF: ETHYLENE RESPONSE FACTOR
FT: FLOWERING LOCUS T
HY5: ELONGATED HYPOCOTYL5
ICE1: Inducer of CBF expression 1
LEA: late embryogenesis abundant
NAC: No Apical Meristem
PIP: Plasma membrane Intrinsic Protein
PP2C: protein phosphatase 2C
PRR: PSEUDO RESPONSE REGULATOR
TIP: Tonoplast Intrinsic Protein
