# Supplementary material for: Comparative Transcriptome Analysis of the Less-Dormant Taiwanese Pear and the Dormant Japanese Pear during Winter Season
Source: PLoS One. 2015 Oct 9;10(10):e0139595. doi: 10.1371/journal.pone.0139595 (PMC4599857; doi:10.1371/journal.pone.0139595)
Supplement: S1 Table — (DOCX) [file pone.0139595.s001.docx]

| Target gene | Primer sequence (5’ to 3’) | Product size (bp) |
| --- | --- | --- |
| Unigene21464 | AGGCATTTAGAGGACCTGACGA | 85 |
|  | GAGCAACCAGCCCCAAA |  |
| Unigene24896 | CAATTCCAGCATACGGAGCA | 95 |
|  | GCAACCACTCAACCCTCACA |  |
| Unigene28589 | TCTCCCGAATCGAAAACAAGA | 150 |
|  | AGAAAAGCCGAAAGCAGAACC |  |
| Unigene50008 | TTGGTTGTTTGGGTGTTGTTG | 135 |
|  | ATGACGGTGGAGCTGATGAA |  |
| Unigene54944 | AGCCTCCGAAAATAGCCTCAC | 134 |
|  | CTCGCTTCCCTTGACTTGCT |  |
| Unigene9700 | CGAAGGGCTCCAGAAAAGG | 84 |
|  | TCCTCTGCCTAACACCCCTAAA |  |
| Unigene44252 | GAGGAGTGAGGAGGAGGAACAA | 105 |
|  | CCATCTCTGCCGTTGGATAAG |  |
| Unigene10919 | TTCGTTTGAGGCGGTGTG | 124 |
|  | ACTATTTCCTTGCGGTGGTTGT |  |
| Unigene11862 | GCTCTTCGTCGTCTTCTTCCTC | 93 |
|  | TCCGTATTCCTCTGTATTGCTTTTC |  |
| Unigene46364 | TTATGCAGCTTTAGAGGAGTGAGG | 100 |
|  | TGAAAGTCCCGAGCCAAAG |  |
| Unigene15251 | GCCCTCACCATCCTCCTTT | 98 |
|  | GATGCTCTACTTCCACTTCAACCTC |  |
| Unigene27973 | GAACCCATCCTTTTATTTTGTCTCC | 125 |
|  | TCGTCTCCGCCAATCCA |  |
| Actin | CAGGCATTCACGAGACCACA | 112 |
|  | TGCCAGGGAACATGGTAGAA |  |

S1 Table.
