# Supplementary material for: Comparative Transcriptome Analysis of the Less-Dormant Taiwanese Pear and the Dormant Japanese Pear during Winter Season
Source: PLoS One. 2015 Oct 9;10(10):e0139595. doi: 10.1371/journal.pone.0139595 (PMC4599857; doi:10.1371/journal.pone.0139595)
Supplement: S2 Table — Z 12 libraries as biological replicates constructed from the floral buds of each sample number. Number of replicates shown in brackets. (DOCX) [file pone.0139595.s002.docx]

S2 Table. ^Z^ 12 libraries as biological replicates constructed from the floral buds of each sample number. Number of replicates shown in brackets.

| Libraries ^Z^ | T1 (1) | T1 (2) | T1 (3) | H1 (1) | H1 (2) | H1 (3) | T2 (1) | T2 (2) | T2 (3) | H2 (1) | H2 (2) | H2 (3) |
| --- | --- | --- | --- | --- | --- | --- | --- | --- | --- | --- | --- | --- |
| Total reads | 66,739,648 | 64,261,636 | 70,111,610 | 70,002,722 | 63,091,218 | 67,431,310 | 33,374,070 | 25,299,258 | 28,828,972 | 29,120,864 | 29,645,050 | 31,857,880 |
| Total trinity transcripts | 73,002 | 79,920 | 79,864 | 81,728 | 77,579 | 79,979 | 69,763 | 65,058 | 66,606 | 64,100 | 66,531 | 66,310 |
| Total trinity components | 49,265 | 53,997 | 53,715 | 54,065 | 51,620 | 52,658 | 49,007 | 45,204 | 46,625 | 45,850 | 47,010 | 46,633 |
| GC percentage | 43.38 | 43.1 | 43.16 | 43.46 | 43.48 | 43.33 | 43.57 | 43.66 | 43.74 | 44.1 | 44.04 | 44.05 |
| Contig N50 | 1,328 | 1,642 | 1,585 | 1,516 | 1,627 | 1,602 | 1,490 | 1,506 | 1,492 | 1,380 | 1,447 | 1,488 |
| Median contig length | 565 | 613 | 602 | 572 | 628 | 627 | 554 | 591 | 567 | 550 | 561 | 582 |
| Average contig | 843.16 | 979.78 | 954.12 | 913.52 | 979.68 | 974.82 | 895.65 | 918.74 | 901.41 | 851.09 | 885.87 | 908.68 |
| Total assembled bases | 61,552,649 | 78,304,128 | 76,199,596 | 74,660,029 | 76,002,909 | 77,965,365 | 62,483,523 | 59,771,677 | 60,039,177 | 54,554,654 | 58,937,771 | 60,254,451 |
| Mapped Reads percentage  (CD-HIT) | 81.6 | 79.7 | 79.1 | 77.7 | 80.8 | 81.3 | 80.8 | 77.0 | 79.3 | 84.0 | 78.0 | 76.6 |
| Mapped Reads percentage  (TGICL) | 73.3 | 70.3 | 70.3 | 72.9 | 67.4 | 71.4 | 68.3 | 68.2 | 65.7 | 70.0 | 66.2 | 65.1 |
